# Increase of Solid Polymer Electrolyte Ionic Conductivity Using Nano-SiO_2_ Synthesized from Sugarcane Bagasse as Filler

**DOI:** 10.3390/polym13234240

**Published:** 2021-12-03

**Authors:** Yatim Lailun Ni’mah, Zakkiyyah Hidayatul Muhaiminah, Suprapto Suprapto

**Affiliations:** Department of Chemistry, Institut Teknologi Sepuluh Nopember, Surabaya 60111, Indonesia; zakiyah.muhaimin@gmail.com (Z.H.M.); suprapto@chem.its.ac.id (S.S.)

**Keywords:** solid polymer electrolyte, PEO, NaClO_4_, Nano-SiO_2_, sodium ion

## Abstract

The synthesize of solid polymer electrolyte (SPE) based on polyethylene oxide (PEO), NaClO_4_ and nano-SiO_2_ was carried out by solution cast technique. Nano-SiO_2_ was synthesized from sugarcane bagasse using sol-gel method. FTIR analysis was carried out to investigate the bonding between nano-SiO_2_ and PEO/NaClO_4_. The morphology of the SPE was characterized using SEM. XRD and DSC analysis showed that SPE crystallinity decreased as nano-SiO_2_ concentration was increased. Mechanical analyses were conducted to characterize the SPE tensile strength and elongation at break. EIS analysis was conducted to measure SPE ionic conductivity. The PEO/NaClO_4_ SPE with the addition of 5% nano-SiO_2_ from sugarcane bagasse at 60 °C produced SPE with the highest ionic conductivity, 1.18 × 10^−6^ S/cm. It was concluded that the addition of nano-SiO_2_ increased ionic conductivity and interface stability at the solid polymer electrolyte-PEO/NaClO_4_.

## 1. Introduction

Battery is an energy storage device that is widely used in today daily life. Currently, lithium-ion batteries have been well developed and have become the frontier in green energy sources [1]. Nevertheless, the natural abundance of lithium (2–7 × 10^−3^ % wt.) in the earth’s crust will become a constraint in its future development [2]. The sodium ion battery is an alternative to replace lithium ion batteries because of its comparable performance and the abundance of sodium in nature [3].

Battery performance is influenced by the material composition that used as its components. One of the battery components is electrolyte. There are two types of electrolytes that can be applied in battery: liquid and solid. However, liquid electrolyte has several disadvantages in the application of the battery. Liquid electrolyte contains volatile and flammable compounds that have been related to battery burning or explosion accidents [4]. Solid polymer electrolyte has been considered as a safer component to be used in batteries, due to its versatility and thermodynamic stability [5]. Thus, solid polymer electrolytes can be used as an alternative to replace liquid electrolytes [6]. 

PEO is one of the polymers that is widely used as a solid electrolyte. This is due to its good electrochemical performance compared to other polyether, co-polymers or PEO-branched electrolytes [2]. PEO form a semi-crystalline phase at room temperature which can limit the ionic conductivity of the solid electrolytes. However, the ionic conductivity of most polymer electrolytes is significantly lower compared to solid oxide electrolytes and liquid electrolytes. Solid polymer electrolyte has not been applied in commercial batteries because of its low ionic conductivity (10^−8^ S/cm at room temperature) and electrochemical stability [7].

PEO that combined with other polymers, plasticizer, fillers, grafting and crosslinking agent have been utilized to improve ionic conductivity of PEO-based solid polymer electrolyte [8]. The solid electrolyte ionic conductivity can be enhanced by increasing the amount of salt in the electrolyte structure, providing a plasticizer with low molecular weight in the polymer matrix [9]. In previous studies on solid electrolyte polymers for sodium ion batteries, NaClO_4_ salt was added as source of sodium ions and plasticizer [2]. Plasticization is the conventional way to reduce crystallinity and increase the amorphous phase of the polymer electrolytes [10]. PEO based solid polymer electrolyte has good flexibility and plasticity as well as good contact ability and interface compatibility with the electrodes [8]. However, it has a crystalline phase at room temperature which can affect its conductivity.

The crystallinity of PEO can be reduced by filler addition. The addition of nano-sized ceramic fillers leads to an enhancement in ionic conductivity while retaining the mechanical strength of the solid polymer electrolyte [10]. The regularity of the PEO molecular chains was disrupted by the addition of filler, so that the crystallinity of PEO was decreased [11]. Inorganic oxide fillers such as alumina (Al_2_O_3_), silica (SiO_2_), titanium oxide (TiO_2_) [12] and fly ash have been studied [13]. The addition of inorganic fillers into the polymer electrolyte film leads to an improvement in the ionic conductivity, from 1.701 × 10^−5^ S/cm to 2.970 × 10^−5^ S/cm (with the addition of Al_2_O_3_) and 3.570 × 10^−5^ S/cm (with the addition of SiO_2_) [14]. The addition of montmorillonite 5% to PPMA produces the highest ionic conductivity of 2.09 × 10^−6^ S/cm [15]. The addition of MgO 6% to PVDF produces a PVDF composite with ionic conductivity of 8.78 × 10^−5^ S/cm [16]. The application of silica nanoparticles as fillers in polymer nanocomposites has drawn much attention, due to improvement in thermal, mechanical, physical and chemical properties [17]. Nano-SiO_2_ as nano filler in the SPE shows some prospective applications [4].

Silica is the second most abundant element in the earth surface, which accounts for approximately 32% of its total weight [18]. SiO_2_ is found in several plants such as in sugarcane bagasse, rice husks and nut grass. Sugarcane bagasse has been used as animal feed, fertilizer production or reused as fuel in sugar factories. However, the ashes are often just thrown away. Hindaprasirt and Rattanasak [19] stated that sugarcane bagasse ash has a rich content of silica, so it can be used as a source of nano-SiO_2_. Nano-SiO_2_ from sugarcane bagasse is used as filler in solid polymer electrolyte because of its high purity. Some impurities of SiO_2_ from sugarcane bagasse were K_2_O, Fe_2_O_3_ and CaO [20]. 

The interaction originating from the SiO_2_ surface can cause stability of the interface between the filler and polymer matrix which in turn will suppress the crystallinity of the polymer and increase the ionic conductivity of solid electrolyte polymers [21]. Based on the background that has been described, a solid polymer electrolyte consisting of PEO/NaClO_4_ with nano-SiO_2_ from sugarcane bagasse as a filler is proposed to reduce PEO crystallinity and increase its ionic conductivity.

## 2. Materials and Methods

### 2.1. Materials

The materials used were sugarcane bagasse as a source of SiO_2_, sodium hydroxide (NaOH, Merck, Darmstadt, Germany, ≥99.0%), Hydrochloric acid (HCl, Merck, Darmstadt, Germany, 37%), sodium percholorate (NaClO_4_, Merck, Darmstadt, Germany, ≥98.0%), acetonitrile (Merck, Darmstadt, Germany, ≥99.0%), polyethylene oxide (PEO, molecular weight 600,000, Sigma Aldrich, Taufkirchen, Germany). All chemicals were purchased from local store in Surabaya Indonesia.

### 2.2. Preparation of Solid Polymer Electrolytes

Polyethylene oxide (PEO) and NaClO_4_ were used as solid polymer electrolytes. PEO and NaClO_4_ were dissolved in 10 mL acetonitrile and stirred for 6 h at 55 °C to obtain homogeneous mixture. A mixture of PEO and NaClO_4_ in ratio of 20:1 were prepared based on a previously published method by Ni’mah et al. [2]. The mixture was molded in a Teflon plate with a diameter of 2 cm and heated for 6 h at 55 °C.

### 2.3. Synthesis of Nano-SiO_2_ from Sugarcane Bagasse

Extraction of nano-SiO_2_ was carried out based on previously published method by Falk et al. [2]. The sugarcane bagasse was washed and dried then cut into smaller size. Sugarcane bagasse was calcined at 650 °C for 3 h. A total of 1 g of sugarcane bagasse ash was washed with 10 mL HCl 1 M and stirred for 1 h at 100 °C. The mixture was filtered and the residue was washed with distilled water for several times. The residue was added with 8 mL NaOH 1 M and stirred at 100 °C for 1 h. The mixture was filtered and the filtrate obtained was sodium silicate. The pH adjustment to pH 7 was carried out for sodium silicate using HCl 1 M so that aquagel was formed. The aquagel that formed was left overnight and dried. The silica obtained was sieved with a 200-mesh sieve to obtain a homogeneous size of silica. The silica was later used as a filler in the solid electrolyte PEO/NaClO_4_.

### 2.4. Preparation of Solid Polymer Electrolytes PEO/NaClO_4_ with Nano-SiO_2_ as Filler

PEO, NaClO_4_ and nano-SiO_2_ were mixed in a Teflon tube and 10 mL of acetonitrile with a ratio of EO: Na 20:1 and nano-SiO_2_ with concentration of nano-SiO_2_ 1, 3, 5 and 7% *w/w* were added. The mixture was stirred for 6 h at 55 °C until a homogeneous mixture was formed. The mixture was then molded in a Teflon plate and dried in an oven at 55 °C for 6 h as in the previous preparation that has diameter of 2 cm and average thickness of 0.2 mm.

### 2.5. Characterization

X-ray Diffraction (XRD) was carried out using Philips X-Pert XRD (Worcestershire, UK) with a Cu-Kα light source (λ = 1.5405 Å, 40 kV and 30 mA). Samples were analyzed in the range of 20 = 5°–60° with a scan rate of 0.5 °/min at room temperature. 

FTIR analysis was carried out using a Shimadzu FTIR-8400 (Columbia, MD, USA) spectrometer with wavenumber ranges from 400 to 4000 cm^−1^ at room temperature.

Thermal analysis of solid polymer electrolytes was carried out using differential scanning calorimetry (DSC) using Perkin Elmer Jade (Shelton, CT, USA) on high pressure gold coated stainless capsules. The sample was molded into capsule shape then put into a glovebox with an argon atmosphere and heated from 25 °C to 100 °C with a heating rate of 10 °C/min.

The Scanning Electron Microscopy (SEM) analysis was used to observe the surface morphology of solid polymer electrolyte. The SEM measurement was conducted at low vacuum and the sample surface was coated with gold-palladium.

The tensile strength was measured to investigate the elongation of solid polymer electrolyte at room temperature. The samples were clamped at both ends and pulled at constant test speed 5 mm/min. Tensile strength, Young’s modulus, elongation strength and yield strength were obtained. 

The ionic conductivity of the polymer electrolyte film was measured using electrochemical impedance spectroscopy (EIS) in which a solid electrolyte was placed between two stainless steels. EIS analysis was carried out using the Metrohm Nova AutoLab B.V tool with a frequency range of 1 MHz-1 Hz and a voltage of 10 mV. The ionic conductivity of the sample is calculated using the following equation [2]:

(1) $$\sigma =\frac{t}{R_{b}A}$$

where σ is ionic conductivity, t is the thickness of film, R_b_ is the resistance and A is the effective film-electrode contact area. The temperature dependence of the ion conductivity was recorded from 30–90 °C.

## 3. Results

### 3.1. Synthesis of Nano-SiO_2_ from Sugarcane Bagasse

**Figure 1:**
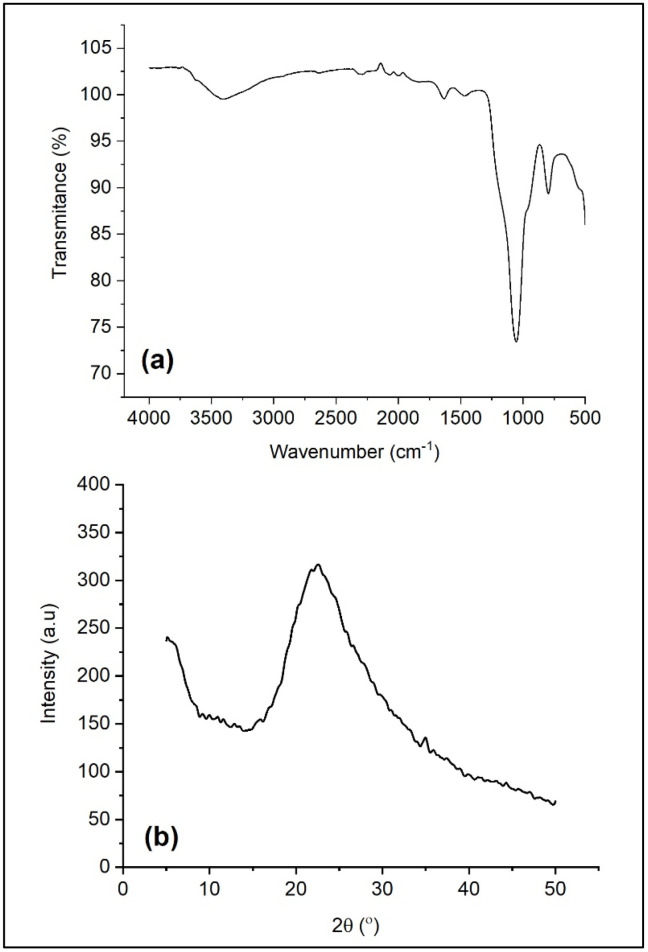
FTIR spectra (**a**) and XRD pattern (**b**) of nano-SiO_2_ from sugarcane bagasse.

Nano-SiO_2_ was synthesized from sugarcane bagasse using sol-gel method based on the method published by Falk et al. [2]. Figure 1a represents the FTIR spectra of nano-SiO_2_ from sugarcane bagasse. The bands at 1200–1000 cm^−1^ and 793 cm^−1^ come from asymmetry and symmetry stretching vibrations of Si-O-Si, respectively. The peak at 1050 cm^−1^ belongs to the SiO-H asymmetry stretching vibration. [22].

Figure 1b represents the XRD diffractogram of nano-silica from sugarcane bagasse which can be used to analyze the degree of crystallinity qualitatively and quantitatively. The broad diffraction peak at 2θ = 22.6° belongs to characteristic peak of amorphous SiO_2_. The crystallite size estimated by the Scherer Equation was 53 nm. The synthesized nano-SiO_2_ was used as a filler in the PEO/NaClO_4_ solid electrolyte polymer. The addition of the nano-sized particles could effectively decrease the crystallinity of the PEO host [23].

### 3.2. Preparation of Solid Polymer Electrolyte

Solid electrolyte polymers were prepared based on the previously reported by Ni’mah et al. [23] with the ratio of PEO and NaClO_4_ = 20:1. The synthesized nano-SiO_2_ was added to the solid polymer electrolyte to decrease the crystallinity of PEO, so that its ionic conductivity increases.

**Figure 2:**
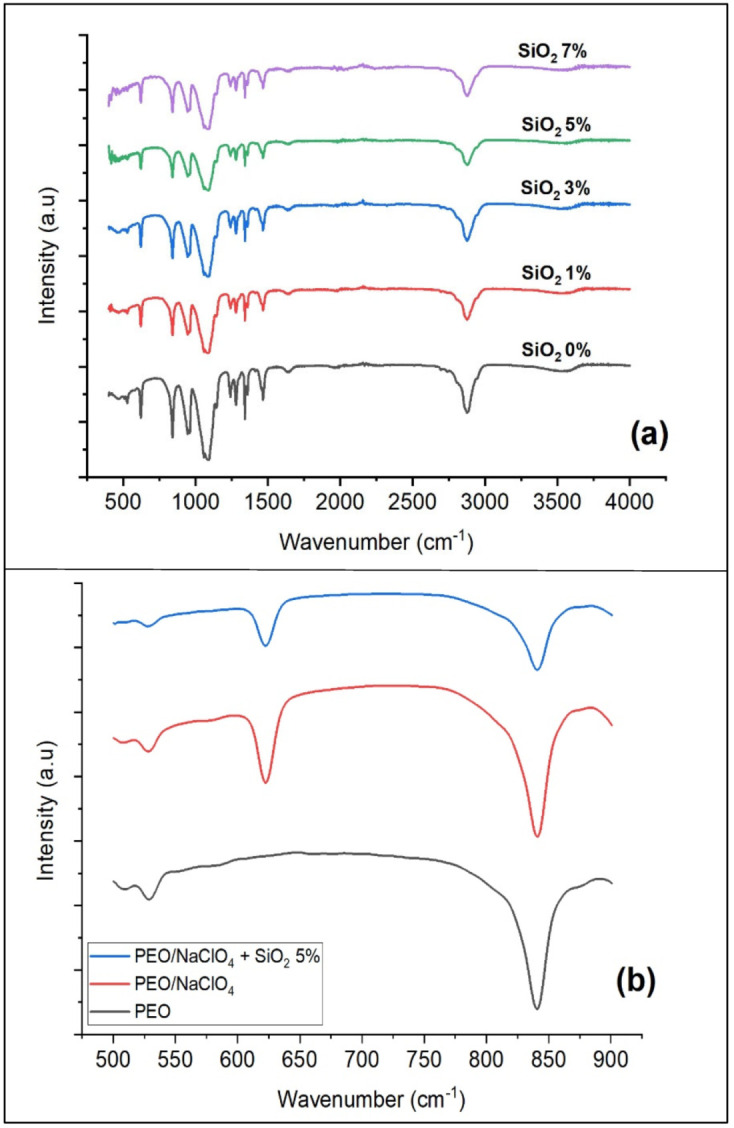
FTIR spectra of solid polymer electrolyte (**a**) PEO/NaClO_4_ + nano-SiO_2_ 0%, 1%, 3%, 5% and 7% (**b**) PEO, PEO/NaClO_4_ and PEO/NaClO_4_ 5% in 500–900 cm^−1^ spectral range.

FTIR spectra of solid polymer electrolyte was used to figure out the interaction between PEO matrix and sodium salt. The peak at wavenumber of 3300–3700 cm^−1^ in Figure 2a was the peak of the O-H vibration bond. The vibration of CH_2_ was observed at the wavenumbers of 2875, 1466, 1359, 1341, 1279, 1241 and 841 cm^−1^. The peaks at 1091 and 1058 cm^−1^ were the peaks of the C-O-C vibration. The peak at wavenumber of 600–650 cm^−1^ was the peak of ClO_4_^−^ that indicates a bond formation between PEO and NaClO_4_, because this peak did not appear in pure PEO as seen in Figure 2b. There were no new peaks upon the addition of nano-SiO_2_, indicating that no new bonds were formed. However, the spectra peaks of the solid polymer electrolyte PEO/NaClO_4_ after addition of nano-SiO_2_ were decreased. This indicated that the nano-SiO_2_ was present in the polymer electrolyte matrix as a different phase and the polymer electrolyte was trapped in the polymer matrix. This also indicates that no significant interaction between electrolyte and polymer was observed [24].

**Figure 3:**
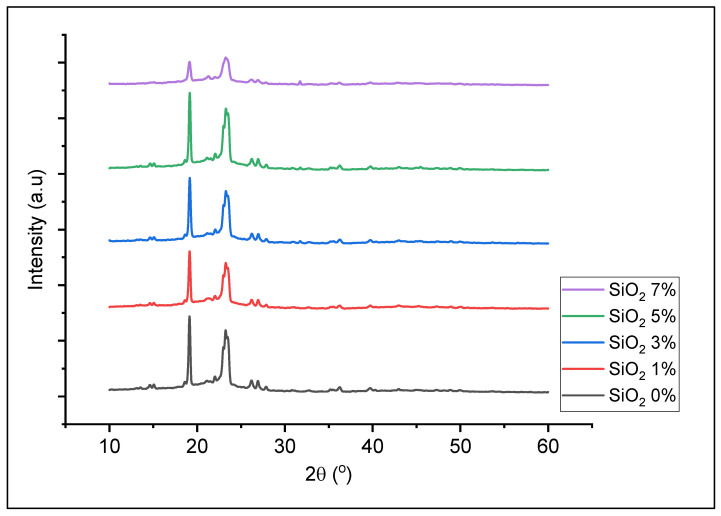
XRD diffractogram of PEO/NaClO_4_ solid polymer electrolyte with 0%, 1%, 3%, 5% and 7% nano-SiO_2_.

XRD diffractograms of PEO/NaClO_4_ solid electrolyte polymer film with different concentrations of nano-SiO_2_ are presented in Figure 3. The peaks at 2θ = 19° and 23° were the characteristics peaks of PEO. The addition of nano-SiO_2_ caused the peak intensity of the solid polymer electrolyte decreased as the concentration of nano-SiO_2_ increased. The addition of nano-SiO_2_ 7% resulted in the lowest peak, indicating that the solid electrolyte polymer had the lowest crystallinity and had highest amorphous phase. The addition of larger amounts of nano-SiO_2_ produce aggregate rather than reducing the degree of crystallinity [4]. This can be attributed to the ion conductivity of PEO/NaClO_4_ with nano-SiO_2_ 7% which decreased to 8.84 × 10^−8^ S/cm. This evident proved that the addition of nano-SiO_2_ to solid electrolyte polymers at a certain level can reduce the crystallinity of PEO, where Na^+^ ions in the film can move freely so that the ionic conductivity increases.

**Figure 4:**
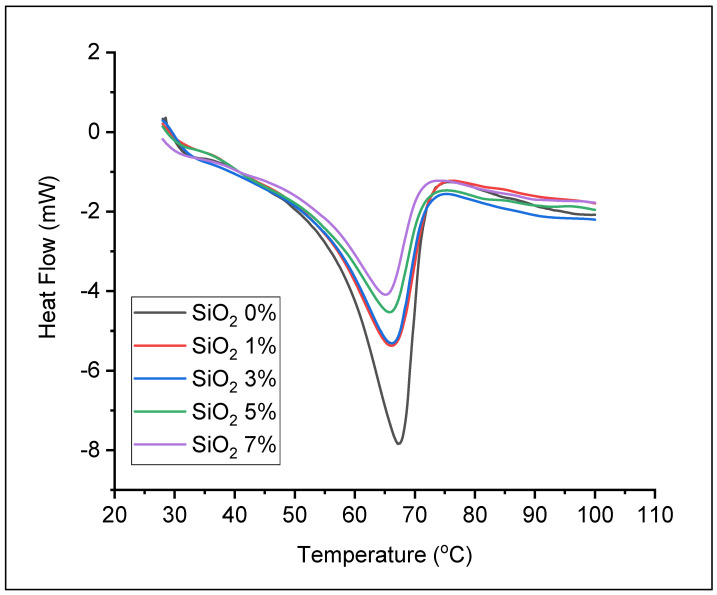
DSC curves of various solid polymer electrolytes PEO/NaClO_4_ + nano-SiO_2_ as filler.

Thermal analysis curve of solid polymer electrolytes using DSC can be seen in Figure 4. The endothermic peak at about 67 °C indicates the melting temperature (Tm) of the sample. The addition of nano-SiO_2_ to the solid electrolyte polymer decreased the endothermic peak and the melting point. The melting points were decreased after the addition of 1% and 3% nano-SiO_2_ to 66 °C and decreased to 65 °C with addition of 5% and 7% nano-SiO_2_. The decrease in melting point is due to the decrease of solid electrolyte polymer film crystallinity with increase of the nano-SiO_2_ content, according to the results obtained by XRD characterization, Figure 3. It was estimated that the nano-SiO_2_ was dispersed homogeneously to fills the space between the PEO chains, thereby preventing or inhibiting PEO crystallization due to the large surface area [25]. 

As mentioned from XRD and DSC analysis, PEO/NaClO_4_ with addition of nano-SiO_2_ decreases the crystallinity and melting point (Tm), confirmed by calculating the relative percentage of crystallinity (X_c_) using the following equation [16]:

(2) $$X_{C}=\frac{\Delta H_{C}}{\Delta H_{P}}$$

where, ΔH_p_ equals to 203 J/g which is the heat enthalpy of composite melting [26], and ΔH_c_ is the heat enthalpy of the samples. The calculated relative percentage of crystallinity and the data obtained from DSC analysis are summarized in Table 1. The value of relative crystallinity percentage decrease after filler addition, so that PEO/NaClO_4_ + nano-SiO_2_ 7% showed the lowest value (17.07%).

**Figure 5:**
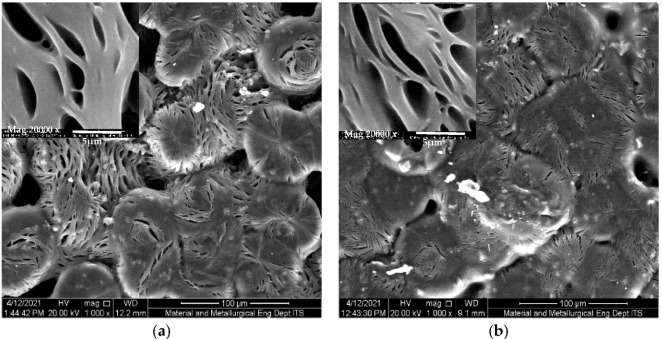
SEM micrographs of solid polymer electrolyte film (**a**) PEO/NaClO_4_ and (**b**) with addition 5% of silica nanoparticle.

SEM micrographs of the solid polymer electrolyte film with/without the filler were shown in Figure 5. The compatibility between the polymer matrix and the inorganic fillers (silica nanoparticles) has great influence on the properties (mechanical, thermal, ionic conductivity and interface stability) of the PEO-based composite electrolytes [27]. Figure 5a shows the morphology for solid polymer electrolyte PEO/NaClO_4_ before the addition of silica nanoparticle. PEO film with the addition of silica nanoparticle shows a smoother polymer film surface, Figure 5b. The porosity of the polymer film increases after the nano-SiO_2_ addition. A smoother SPE surface means the ions can move freely in the electrolyte, thus the conductivity enhancement was produced [14].

Tensile strength and elongation of solid polymer electrolyte polymer was presented in Table 2. It can be seen that tensile strength of solid polymer electrolyte decreased from 0.31 MPa before addition of nano-SiO_2_ 5% filler to 0.29 Mpa. In addition, the percentage of elongation also decreased from 270% to 260% after addition of silica nanoparticles to the film. This is indicated that the addition of silica nanoparticles reduces the flexibility of the film.

The ionic conductivity of solid polymer electrolyte increased after the addition of NaClO_4_ salt and nano-SiO_2_ as fillers, as calculated by Equation (1). The ionic conductivity of PEO solid electrolyte polymer was 1.78 × 10^−9^ S/cm and increased to 4.84 × 10^−8^ S/cm after the addition of NaClO_4_. The highest ion conductivity of solid electrolyte polymer was 1.10 × 10^−6^ S/cm obtained by the addition of 5% nano-SiO_2_ at room temperature. However, the ionic conductivity decreased to 8.84 × 10^−8^ S/cm after 7% of nano-SiO_2_ was added to the solid electrolyte polymer. This increase in ionic conductivity is due to decrease in the degree of crystallinity and generates more amorphous region [4]. The steric inhibition effect of nano-SiO_2_ means a solid polymer electrolyte ion transport occurs via intra-chain and inter-chain charge transfer in the amorphous phase [14]. The amount of nano-SiO_2_ added to the polymer solid electrolyte affects its ionic conductivity as the distance between the fillers decreases, causing blocking effect, so that it is more difficult for ions to move [28]. Another possibility that occurs is that free ions form ion pairs or aggregates that can reduce the number of sodium ions [24].

**Figure 6:**
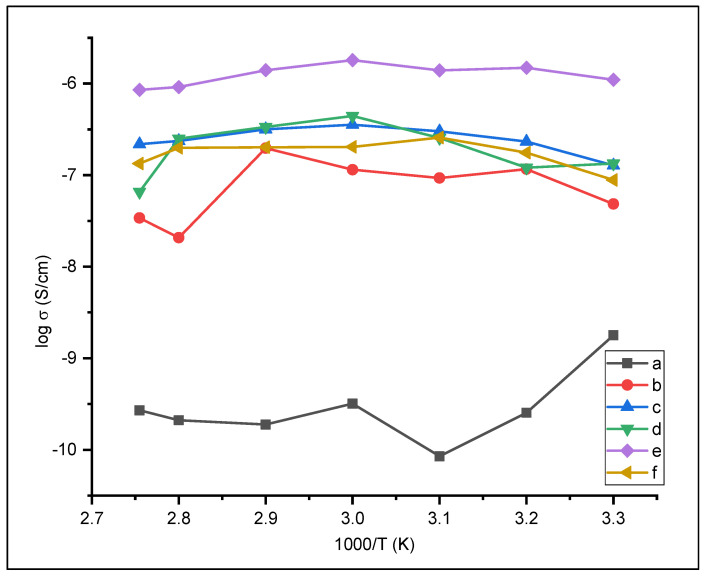
Temperature-dependent of ionic conductivity (**a**) PEO (**b**) PEO/NaClO_4_ (**c**) PEO/NaClO_4_ + nano SiO_2_ 1% (d) 3% (e) 5% (f) 7%.

In general, the ionic conductivity of solid polymer electrolyte increases with the increase of temperature. This is due to movement of charge in the polymer chain in the amorphous phase increases with increasing temperature [25]. Figure 6 shows the temperature-dependent ionic conductivity of solid polymer electrolyte. The highest temperature-dependent ionic conductivity of solid electrolyte polymers was 1.8 × 10^−6^ S/cm with the addition of 5% nano-SiO_2_ at 60 °C. The temperature-dependent on the ionic conductivity of solid electrolyte polymer can be related to the data obtained from the DSC result, where the ion conductivity will decrease at a temperature higher than SPE melting point.

**Figure 7:**
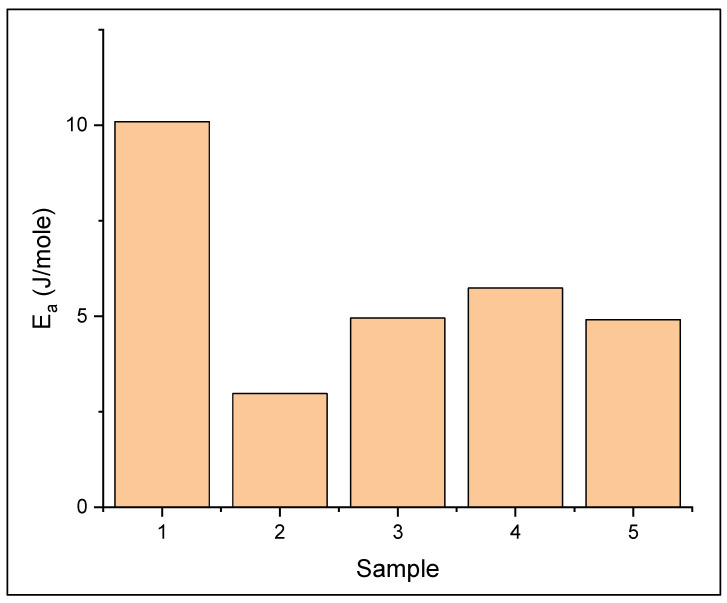
Activation energy values of (1) PEO/NaClO_4_ (2) PEO/NaClO_4_ + nano SiO_2_ 1% (3) 3% (4) 5% (5) 7%.

Furthermore, the activation energy (E_a_) are obtained using the Arrhenius model:

(3) $$\sigma =\sigma_{0}\exp\left(-\frac{E_{a}}{\mathrm{RT}}\right)$$

where σ, E_a_, σ_0_, T and R represent the ionic conductivity, activation energy, pre-exponential factor, a temperature factor and the ideal gas constant, respectively. The activation energy value of solid polymer electrolyte is shown in Figure 7. Solid polymer electrolyte PEO/NaClO_4_ before addition of nano-SiO_2_ shows higher activation energy value than after the addition of nano-SiO_2_. The lower E_a_ demonstrates that after addition of nano-SiO_2_, the ion movement in the solid polymer electrolyte needs less energy than before the addition, indicating a higher conductivity [29].

## 4. Conclusions

Nano-SiO_2_ has been successfully synthesized from sugarcane bagasse using sol-gel method with the average size of 53 nm. The SPE was fabricated from PEO, NaClO_4_ and nano-SiO_2_ by casting method. XRD diffractogram at 2θ = 19° and 23° shows that the crystallinity of the SPE decreased as nano-SiO_2_ increased which was confirmed by DSC melting point at 67 °C. FTIR spectra indicates that peak at wavenumbers of 600–650 cm^−1^ were the peaks of ClO_4_^−^, which intensity was decreased as the number of nano-SiO_2_ increased. SEM characterization showed that the addition of nano-SiO_2_ increased the number of pores in the surface of SPE. The addition of nano-SiO_2_ decreased the tensile strength and elongation at break. The PEO/NaClO_4_ + 5% of SiO_2_ solid polymer electrolyte has the highest ionic conductivity of 1.8 × 10^−6^ S/cm at 60 °C.

## Figures and Tables

**Table 1:** Values of heat enthalpy melting (ΔH_m_) and percentage of crystallinity (X_c_) of PEO/NaClO_4_ + nano-SiO_2_ as filler.

Sample	Tm (°C)	ΔH_m_ (J/g)	X_c_ (%)
SiO_2_ 0%	67.17	68.48	33.73
SiO_2_ 1%	66.17	46.24	22.78
SiO_2_ 3%	66.17	45.72	22.52
SiO_2_ 5%	65.83	38.80	19.11
SiO_2_ 7%	65.17	34.65	17.07

**Table 2:** Tensile strength and % elongation of PEO/NaClO_4_ and PEO/NaClO_4_ + nano-SiO_2_ as filler.

	Tensile Strength (MPa)	% Elongation
PEO/NaClO_4_	0.31	270
PEO/NaClO_4_ + nano-SiO_2_ 5%	0.29	260

## Data Availability

Not applicable.
